# Time to move beyond risk assessment models for ambulatory cancer-associated thrombosis

**DOI:** 10.1016/j.rpth.2025.102991

**Published:** 2025-07-30

**Authors:** Karlyn A. Martin, Chris Holmes

**Affiliations:** Division of Hematology/Oncology, Larner College of Medicine, University of Vermont, Burlington, Vermont, USA

Ralph Waldo Emerson is often credited with saying, “Build a better mousetrap, and the world will beat a path to your door.” But what if despite building successfully better mouse traps with incremental advances, no one uses a trap at all? This is the failure of venous thromboembolism (VTE) prophylaxis in ambulatory patients starting systemic chemotherapy. Despite numerous validated risk models and support from guideline recommendations, clinical uptake of VTE prevention interventions remains disappointingly low. Medicine increasingly realizes what entrepreneurs have long known: that product success requires real-world utility and adoption, and evidence alone is insufficient without meaningful implementation.

The association between malignancy and VTE has been recognized for over a century and a half. As early as the 1860s, Dr Armand Trousseau described the association of thrombosis with cancer and subsequently recognized “Trousseau’s syndrome” in himself. With time, studies turned to identifying risk factors and developing predictive models for cancer-associated thrombosis. In 2008, Khorana et al. [1] performed a secondary analysis of prospective observational data for a study on chemotherapy-induced neutropenia. Through this analysis, he developed and validated the Khorana Score, which stratifies patients according to their risk of cancer-associated thrombosis based on 5 clinical variables. Since then, numerous risk assessment models for cancer-associated thrombosis have been published, aiming to enhance prediction accuracy (Figure) [[1], [2], [3], [4], [5], [6], [7], [8], [9], [10], [11], [12], [13], [14], [15], [16], [17], [18], [19], [20], [21], [22]]. The 2010 Vienna Cancer And Thrombosis Study modification added D-dimer and P-selectin to the Khorana Score [2]. In 2012, the Prophylaxis of thromboembolism during chemotherapy score incorporated platinum and gemcitabine to the Khorana Score [3]. Other risk prediction models include the 2013 CONKO [4], the 2017 ONKOTEV [5] and Comparison of methods for thromboembolic risk assessment with clinical perceptions and awareness in real life patients-cancer associated thrombosis scores [6], the 2018 Vienna Cancer and Thrombosis Score [7], and the Electronic Health Record-Cancer Associated Thrombosis score in 2023 [8]. Still others incorporate genetic components, such as the 2018 Thrombo inCode-Onco [9] and 2023 ONCOTHROMB [10] scores. This list is not exhaustive, nor does it include numerous studies validating the scores and comparing them across various populations.

**Figure:**
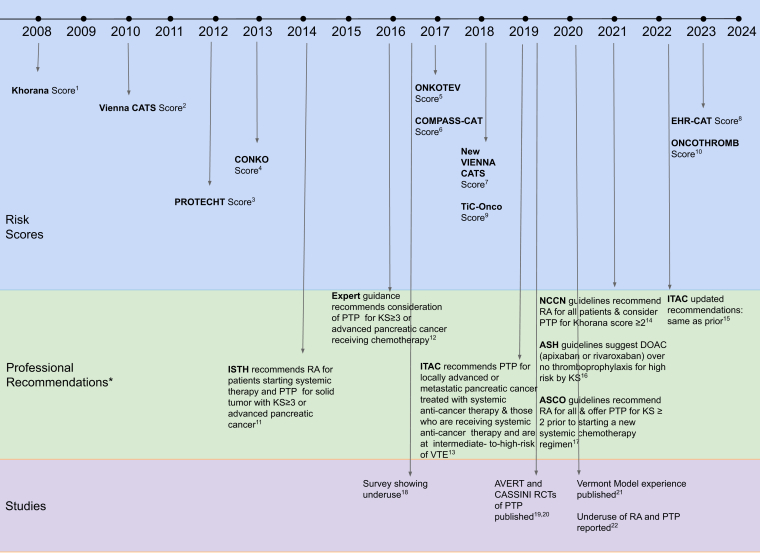
Timeline of risk scores, recommendations, and key studies of venous thromboembolism prevention for ambulatory patients with cancer. ∗Recommendations are for primary prophylaxis for ambulatory patients. DOAC, direct oral anticoagulant; KS, Khorana Score; PTP, primary thromboprophylaxis; RA, risk assessment; RCT, randomized controlled trial; VTE, venous thromboembolism.

For more than a decade, expert guidance and professional society guidelines have recommended performing risk assessments for ambulatory patients with cancer starting chemotherapy and consideration of primary prophylaxis for high-risk patients (Figure). In 2014, the International Society on Thrombosis and Haemostasis first published guidance recommending VTE risk assessment for all ambulatory patients with cancer starting systemic therapy and consideration of anticoagulation thromboprophylaxis for patients with a solid tumor and Khorana Score ≥3 or advanced pancreatic cancer [11]. In 2016 and 2019, international experts in the field authored guidance on prevention of cancer-associated thrombosis, echoing the International Society on Thrombosis and Haemostasis recommendations [12,13]. After randomized trials of targeted thromboprophylaxis for selected high-VTE risk patients [19,20], guidelines from National Comprehensive Cancer Network, American Society of Clinical Oncology, American Society of Hematology, and International Initiative on Thrombosis and Cancer endorsed risk assessment for all ambulatory patients and thromboprophylaxis for high-risk patients [[13], [14], [15], [16], [17]].

Presumably, the objective of continuously refining risk prediction models is to identify patients who could benefit from interventions to reduce their risk of thrombosis. Yet, despite over 2 decades of published models, each offering incremental improvements, alongside repeated recommendations from professional societies and expert guidance, there is little evidence that that these practices have been meaningfully adopted into clinical practice [18,22,23]. The recent validation of 6 risk assessment models in the ViennaCATS cohort speaks to this failure; despite excluding patients on anticoagulation, 806 patients with cancer starting chemotherapy were successfully enrolled from 2019-2024 and followed for 6 months, suggesting high-VTE risk patients were not started on thromboprophylaxis during the study period [24]. We *can* build a better mouse trap, but *should* we? Should efforts continue to refine risk models, if no one is *using the mouse trap*? This is the failure of VTE prevention in ambulatory patients with cancer.

The field has focused on developing and validating newer and better models but has lost sight of the fact that uptake in clinical practice is almost nonexistent. Instead of continuing to attempt a “better mouse trap,” it is necessary to shift toward understanding why barriers exist to the uptake of the risk models into clinical practice. The delayed uptake is not unique to CAT prevention; there are data to suggest it can take 20 years to move evidence into clinical practice [25,26]. Only by targeted study to understand these barriers, followed by development of dedicated implementation strategies to address these barriers, might uptake into clinical practice be improved. Data are emerging on barriers to use and implementation strategies that could facilitate adoption of evidence-based recommendations into clinical practice [21,27,28]. Barriers identified include oncologists’ limited awareness of evidence supporting VTE prevention strategies, overburdened clinicians with too little time, and prioritization of cancer care over VTE prevention. Implementation strategies identified include adapting the electronic health record to provide clinical decision support, providing clinician education to address insufficient awareness of evidence behind the interventions, and using audit and feedback [[27], [28], [29]].

In addition, only by implementing strategies for VTE prevention into clinical practice will implications in “real-world” patients and settings be revealed. Factors affecting patient outcomes that are unidentified or underrecognized by risk assessment models may emerge when using VTE prevention in the real world. In addition, unique barriers across various settings may emerge. For example, the University of Vermont’s program for VTE prevention in ambulatory cancer, a multidisciplinary approach that includes referral to thrombosis specialists, showed success in improving VTE risk assessment, VTE education, and rates of thromboprophylaxis prescriptions in high-risk patients [21]. However, when attempting to translate the Vermont Model into a community-based setting without thrombosis specialists, rates of prophylaxis fell significantly compared with those in the academic setting [30]. These critical data highlight the need to study diverse settings in order to optimize the implementation into clinical practice of evidence-based VTE prevention strategies for ambulatory patients with cancer.

Until we bridge the implementation gap, new risk assessment models are vulnerable to becoming academic exercises rather than tools that impact real people at risk of VTE. We don’t need more mouse traps; we need to *use* the mouse traps. Our patients’ outcomes depend on it.

## Author contributions

All authors substantially contributed to the (1) the conception and design of the study, or acquisition of data, or analysis and interpretation of data; (2) drafting the article or revising it critically for important intellectual content; and (3) final approval of the version to be submitted.
